# Phenotypic and Genotypic Characterization of Methicillin Resistance in *Staphylococci* Isolated from an Egyptian University Hospital

**DOI:** 10.3390/pathogens12040556

**Published:** 2023-04-05

**Authors:** Marwa A. Abdelwahab, Wesam H. Amer, Dalia Elsharawy, Reham M. Elkolaly, Rehab Abd El Fattah Helal, Dina Ahmed El Malla, Yomna G. Elfeky, Hebatallah A. Bedair, Rania S. Amer, Marwa E. Abd-Elmonsef, Marwa S. Taha

**Affiliations:** 1 Department of Medical Microbiology and Immunology, Faculty of Medicine, Tanta University, Tanta 31527, Egypt; 2 Department of Chest Diseases, Faculty of Medicine, Tanta University, Tanta 31527, Egypt; 3 Department of Anathesia, Surgical Intensive Care, and Pain Medicine, Faculty of Medicine, Tanta University, Tanta 31527, Egypt; 4 Department of Pediatrics, Faculty of Medicine, Tanta University, Tanta 31527, Egypt; 5 Department of Clinical Pathology, Faculty of Medicine, Tanta University, Tanta 31527, Egypt

**Keywords:** *mecC*, *mecA*, MRSA, OS-CoNS, PCR, VITEK2

## Abstract

Methicillin-resistant in *Staphylococci* is a serious public health issue. It is mostly encoded by the *mecA* gene. The *mecC* gene is a new *mecA* analog responsible for resistance to methicillin in some *Staphylococcal* clinical isolates. This *mecC* gene is still underestimated in Egypt. The aim of the current study was to detect *mecA and mecC* genes in clinical *Staphylococci* isolates from a tertiary care university hospital in Egypt compared to the different phenotypic methods. A total of 118 *Staphylococcus aureus* (*S. aureus*) and 43 coagulase-negative *Staphylococci* (CoNS) were identified from various hospital-acquired infections. Methicillin resistance was identified genotypically using the PCR technique and phenotypically using the cefoxitin disc diffusion test, oxacillin broth microdilution and the VITEK2 system in all *Staphylococcal* isolates. The *mecA* gene was detected in 82.2% of *S. aureus* and 95.3% of CoNS isolates, while all of the isolates tested negative for the *mecC* gene. Interestingly, 30.2% of CoNS isolates showed the unique character of inducible oxacillin resistance, being *mecA*-positive but oxacillin-susceptible (OS-CoNS). The dual use of genotypic and phenotypic methods is highly recommended to avoid missing any genetically divergent strains.

## 1. Introduction

Methicillin-resistant *Staphylococcus aureus* (MRSA) is a common pathogen capable of producing a wide variety of clinical illnesses [1]. The first report of methicillin resistance in *Staphylococcus aureus (S. aureus)* was published in 1961 [2]. Methicillin resistance has also increased among coagulase-negative *Staphylococci* (CoNS) [3].

The emergence of antibiotic-resistant strains among these *Staphylococci* raises concerns and restricts the number of antimicrobials available for the treatment of these infections [2]. MRSA is one of the most common causes of infections acquired in hospitals. Healthcare-associated MRSA (HA-MRSA) infections are a substantial burden on the healthcare system because of the increased morbidity and extra costs associated with extended hospital stays, as well as higher fatalities than those caused by methicillin-susceptible *S. aureus* (MSSA) [4].

MRSA strains carry a unique and transmissible genetic component known as *Staphylococcal* cassette chromosome *mec* (SCCmec) that harbors the *mecA* gene at the 3′ end of a chromosomal open reading frame named orfX. It encodes a penicillin-binding protein (PBP2a) with a reduced affinity for beta-lactam antibiotics. Consequently, these strains are resistant to all beta-lactam antibiotics, with the exception of fifth-generation cephalosporins [5,6,7].

In 2011, a novel SCCmec—type XI, carrying another mec homolog called *mecC*—was discovered in *S. aureus* [8]. *mecC* shares approximately 70% nucleotide sequence identity with the classical *mecA* gene, causing false negative outcomes when using molecular methods to detect MRSA [9]. PBP2c is the altered PBP encoded by the chromosomal gene *mecC* [10]. It only shows a 63% amino acid homology to PBP2a [11]. There are currently thirteen types of SCCmec known, along with several deletion variants, composites and irregular components. [12,13].

Interestingly, PBP2c’s attachment affinity for oxacillin is four times higher than that of PBP2a. As a result, *mecC*-harboring MRSA demonstrated low-level resistance to β-lactamases [11,14]. Previous studies on *mecC* reported minimum inhibitory concentrations (MICs) of 0.75 to 32 μg/mL for oxacillin and 4 to 64 μg/mL for cefoxitin [11,15]. This low-level resistance exhibited by *mecC*-harboring strains leads to them being misdiagnosed as methicillin-susceptible *S. aureus* when using phenotypic methods, and when treated with β-lactam antibiotics can lead to highly resistant strains [16].

The *mecC*-harboring MRSA has been isolated from a wide variety of host species, including humans, wildlife, livestock and pets from many European countries [8]. In contrast, data about the existence of the *mecC* gene in other *Staphylococcal* species are limited. Only two previous studies have been conducted on the prevalence of *mecC*-gene-harboring MRSA in Egypt [17,18]. Moreover, no previous studies have been conducted on the prevalence of CoNS carrying the *mecC* gene in Egypt, and there are no data about the prevalence of *mecC*-harboring *Staphylococci* in the University hospitals where our study was conducted. Therefore, this study aimed to investigate the presence of *mecC*-harboring Staphylococci isolated from patients suffering from hospital-acquired infections (HAIs) in Tanta University Hospitals.

## 2. Materials and Methods

### 2.1. Patients

This cross-sectional study included 500 patients admitted to different clinical departments, including the Pediatric, Chest, Internal Medicine, and Intensive Care Units of Tanta University Hospitals. Samples were collected over the course of a year, from March 2021 to March 2022.

Inclusion criteria:

- Patients have signs of infection that developed after 48 h of admission.
- Patients show an unsatisfactory response to empirical antibiotic treatment.

Exclusion criteria:

- Patients have infections that develop less than 48 h after admission (community-acquired infection).

Culture-positive specimens taken from sterile sites, e.g., blood and cerebrospinal fluid (CSF), were directly defined as infection. Positive cultures from patients’ sputum, urine, and surgical wound sites were also defined as infection, according to the US Centers for Disease Control and Prevention (CDC) and the National Health Care Safety Network (NHSN) [19,20]. All cases defined as infection gave positive culture results from 48 h after admission.

### 2.2. Bacterial Isolation

Different clinical specimens, including blood, CSF, urine, wound, and sputum, were collected from hospitalized patients admitted to Tanta University Hospitals and transported as soon as possible to the Laboratory of the Microbiology Department for further processing. The samples were first codified, and blood specimens were processed using a qualitative automated culture system (BacT/ALERT 3 D 60, bioMérieux, Marcy-l’Etoile, France) [21]. Positive blood cultures were cultured on BacT/ALERT, and the other collected specimens were cultured on MacConkey’s agar, brain–heart infusion agar and blood agar (Oxoid, England) and incubated at 37 °C for 24–48 h for further identification. Positive growth was observed for colony morphology and Gram stain. Suspected Staphylococci were further identified by biochemical reactions, slide and tube coagulase test, subcultured on mannitol salt agar (Oxoid) and incubated at 37 °C for 24–48 h. Species identification was performed by an automatic VITEK2 system for Gram-positive identification (bioMérieux, France) according to the manufacturer’s instructions. The members of the *S. aureus* clonal complex (*S. argenteus* and S. schweitzeri) were not distinguished from *S. aureus* in our research. Isolates were stored at −80 °C for further antibiotic susceptibility testing and molecular study.

### 2.3. Antibiotic Susceptibility

All *Staphylococcal* isolates were tested for antibiotic susceptibility by the following methods: (i) Kirby-Bauer disk diffusion method using the following antimicrobials discs: penicillin (P) 10 U., cefoxitin (FOX) 30 μg, vancomycin (VA) 30 μg, gentamicin (CN) 10 μg, erythromycin (E) 15 μg, tetracycline (TE) 30 μg, ciprofloxacin (CIP) 5 μg, clindamycin (DA) 2 μg, trimethoprim-sulfamethaxole (SXT) 25 μg, chloramphenicol (C) 30 μg, rifampin (RD) 5 μg, and linezolid (LZD) 30 μg (Oxoid, UK). We interpreted the result of the susceptibility based on the Clinical and Laboratory Standard Institute guidelines (CLSI M100-S29) [22], where FOX inhibition zones of ≤21 mm for *S. aureus* and ≤24 mm for CoNS were considered resistant. (ii) Oxacillin broth microdilution method (BMD) (Sigma-Aldrich, Germany) according to CLSI guidelines [22,23], where MICs were interpreted to be resistant if (≥4 μg/mL) for *S. aureus* and (≥0.5 μg/mL) for CoNS. (iii) VITEK2 system (bioMérieux, France) with the Gram-positive susceptibility panel AST-67 according to the manufacturer’s instructions. The multiple antibiotic resistance (MAR) index of each isolate was estimated according to Tambekar et al.’s method [24].

### 2.4. Molecular Study 

DNA was extracted from all *Staphylococcal* isolates using the QIAamp DNA Mini Kit (Qiagen, Hilden, Germany). The existence of *mecA* and *mecC* genes was detected by conventional polymerase chain reaction (PCR) assay [15,25]. The used primers are shown in Table S1 in the Supplementary section.

Control strains of *S. aureus* American Type Culture Collection (ATCC) 43300 for *mecA* positive, ATCC 25923 for *mecA* negative, and National Collection of Type Cultures (NCTC) 13552 for mecC positive were used as a control for all used tests.

### 2.5. Statistical Analysis

All data were analyzed using the SPSS, Version 26 (IBM Corp, Armonk, NY, United States, 2019). Categorical data were represented as numbers and percentages. The chi-square test was applied to investigate the association between the categorical variables. Alternatively, Monte Carlo or Fisher’s exact correction tests were applied when more than 20% of the cells had an expected count of less than five. Quantitative data were expressed as a range (minimum and maximum), mean and standard deviation. Student’s *t*-test was used to compare two groups in terms of sensitivity, specificity, PPV, NPV and accuracy for agreement between PCR *mecA* and different tests. The significance of the obtained results was judged at the 5% level.3.

## 3. Results

### 3.1. Patient Characteristics

Basic characteristics of the patients infected with the isolated *Staphylococci* are shown in Table 1, regarding age, gender, and included samples. A total of 500 clinical samples were taken from 500 patients admitted to different clinical departments at our hospital, from which 553 isolates were detected, including 210 Gram +ve isolates, 307 Gram −ve isolates and 36 *Candida* species. *S. aureus* was isolated from 118 patients, while CoNS were isolated from 43 patients. Isolates other than *Staphylococci* are displayed in Supplemental Table S2 in the Supplementary section.

There were statistically significant differences between the isolated *Staphylococcal* species regarding different sample types. CoNS were significantly isolated from CSF, while *S. aureus* was significantly isolated from both sputum and wound.

### 3.2. Distribution of Isolated Staphylococci along the Study Period

All the isolated *Staphylococci* were scheduled according to the time of isolation and the number of collected samples. The number of *Staphylococci* was collectively recorded for each season (Table 2). The isolated *Staphylococci* reached its highest in summer (35.8%), while it was significantly lower in winter (15.3%) when compared with the other seasons (*p* < 0.05) (Table 2).

### 3.3. Distribution of CoNS Species Isolated from Different Clinical Samples

This study included 553 isolates from 500 patients admitted to Tanta University Hospitals, Egypt. Of these, 161 isolates (29.1%) were *Staphylococci*, of which the most frequent species were *S. aureus* 118/161 (73.3%), and the remaining 43/161 (26.7%) were CoNS, represented as follows: *S. epidermidis*, 17 (39%); *S. haemolyticus*, 16 (37%); *S. hominis*, 5 (12%); and *S. saprophytic*, 5 (12%). Moreover, *S. epidermidis,* followed by *S. hominis,* were significantly isolated from blood samples. At the same time, both *S. epidermidis* and *S. haemolyticus* were significantly isolated from CSF samples. *S. saprophyticus* represented the most frequently isolated CoNS species from urine samples (Table 3).

### 3.4. Antibiotic Susceptibility Patterns among Staphylococcal Isolates Detected by Disc Diffusion Method

Regarding *S. aureus*, overall, 100% of isolates exhibited resistance to penicillin and cefotaxime. We observed resistance to chloramphenicol as the next highest among 75.4% of the isolates, followed by erythromycin (68.4%). Furthermore, 58.4% and 56.8% of the isolates exhibited resistance to doxycycline and ciprofloxacin, whereas 32.2% and 28% were resistant to gentamycin and trimethoprim–sulfamethoxazole, respectively. We found the lowest resistance rates corresponded to tetracycline (17%), followed by rifampicin (16%). Vancomycin and linezolid were 100% susceptible.

Regarding CoNS, the highest resistance rates to penicillin and cefoxitin were observed in *S. haemolyticus* (87%), followed by *S. epidermidis* (58.8%), while *S. saprophyticus* and *S.hominis* showed the same lowest resistance rate (40%). All CoNS isolates showed 100% susceptibility to vancomycin and linezolid.

### 3.5. Antimicrobial Resistance Patterns among S. aureus Isolates

The antimicrobial resistance patterns of the *S. aureus*-resistant isolates (n = 118) were grouped according to the number and type of the tested antimicrobials with resistant profiles. In general, multiple antimicrobial resistances were common among the tested isolates, where *S. aureus* exhibited 35 antimicrobial resistance patterns. Moreover, *S. aureus*-tested isolates showed very heterogeneous resistance patterns. Based on the antimicrobial resistance patterns of these isolates, MAR index values were calculated (ranging from 0.16–0.83) in Table 4.

### 3.6. Prevalence of Methicillin Resistance among Staphylococcal Isolates Detected by Phenotypic Methods

Regarding cefoxitin DD, resistance was detected in all *S. aureus* isolates (118/118, 100%) and 28/43 (65.1%) of CoNS isolates. Using the oxacillin BMD method, all *S. aureus* isolates were found to be resistant, with MICs ranging from 4 μg/mL to ≥ 512  μg/mL, while 36/43 (83.7%) of CoNS were resistant, with MICs ranging from 0.5  μg/mL to 512 μg/mL.

### 3.7. Prevalence of Methicillin Resistance among Staphylococcal Isolates Detected by Genotypic Method (PCR)

None of the *Staphylococcal* isolates carried the *mecC* gene, while 97/118 (82.2%) of *S. aureus*, 16/17 (94%) of *S. epidermidis*, 15/16 (93.7%) of *S. hemolyticus*, 5/5 (100%) of both *S. hominis* and *S. saprophyticus* isolates were found to carry *mecA* gene (Table 5).

### 3.8. Sensitivity and Specificity of Cefoxitin DD, VITEK2 Cefoxitin, VITEK2 Oxacillin, and BMD Oxacillin in Detecting Methicillin Resistance Compared to the Genotypic Method (mecA PCR)

Regarding *S. aureus,* methicillin resistance detected by VITEK2 oxacillin showed only 85.57% sensitivity and 88% accuracy, whereas the other phenotypic methods showed 100% sensitivity with 82% accuracy.

Concerning CoNS, methicillin resistance tested by VITEK2 cefoxitin showed the highest sensitivity (97.56%), followed by VITEK2 oxacillin (90.24%), while cefoxitin DD showed the lowest sensitivity. Moreover, all tests showed 100% specificity, with VITEK2 cefoxitin having the highest accuracy (97.67%). (Table 6).

## 4. Discussion

MRSA is one of the most significant microorganisms associated with hospital infections globally. It is no longer confined to ICUs, burn units, and specialized medical facilities but has also extended to less critical departments, posing significant problems to hospital infection control [26]. Methicillin resistance in *Staphylococci* is based on the production of mutated penicillin-binding proteins with a reduced affinity for beta-lactam antibiotics. These proteins are encoded by various mec genes (*mecA* or *mecC*), of which *mecA* is the most prevalent and frequent [19]. This study aimed to investigate the prevalence of both *mecA* and *mecC* genes among isolated *Staphylococci.*

In the current study, *Staphylococcus* spp. isolates were recovered from 32.2% of the processed clinical samples. The isolated *Staphylococci* showed statistically significant seasonal variations; the number of isolates reached its highest in summer and was significantly lower in winter when compared to the other seasons. Similar to ours are the results of Casson et al. [27], who found that during late spring and early summer, MRSA incidence reached its peak, while it troughed during late fall and early winter; their findings may be attributed to the increased rate of intravenous vancomycin usage during periods with high MRSA incidence, with a possible association with antimicrobial usage. However, the seasonality of bacterial illnesses frequently links rising temperatures to rises in infection rates. Notably, seasonality in hospitals is reported by a study on *S. aureus* infections [28].

In the current study, 39% of MRSA isolates were recovered from blood, followed by wound swabs and sputum (38% and 12% of isolated MRSA, respectively). Our findings were to some extent similar to Shebl et al. [18], who isolated 50% of MRSA from blood, followed by wounds and sputum. Contrary to our results, Khan et al. [29] isolated 62% of MRSA from pus, 14% from urine and only 9% isolated from blood.

Among MRSA isolates, we detected 100% susceptibility to vancomycin and linezolid, in accordance with the results of Girgis et al. [29], who detected 100% susceptibility to vancomycin. Additionally, Khan et al. and Al-Zoubi et al. [30,31] reported 96% and 96.5% susceptibility to linezolid, respectively. On the other hand, we detected 100% resistance to penicillin, which was comparable with the results described in other surveys conducted in various governorates in Egypt [32,33]. These results suggest that *S. aureus* infections in Egypt can no longer be treated with this drug. Additionally, high resistance to chloramphenicol was detected (75.4%), followed by erythromycin (68.4%), doxycycline (58.4%), and ciprofloxacin. This was in alignment with various studies worldwide [29,34,35,36,37], whereas our pattern of resistance was lower than that reported for MRSA by Karami et al. [38].

The first identification of *mecC*-harboring MRSA was in southwest England, isolated from a tank milk specimen [39]. Since this discovery, *mecC* has been widely detected among livestock and wild animals [40]. However, its distribution in humans is still low [27,41] and has mainly been reported in Europe so far [9,14,42,43]. According to earlier research, the nations with the highest *mecC* concentrations in MRSA isolates were the UK and Denmark [11,44,45]. The *mecC* gene was not detected in any of our PCR-tested MRS isolates. Similarly, the absence of the *mecC* gene in MRS isolates from human samples was reported by several recent studies worldwide [17,46,47,48,49,50]. On the other hand, the *mecC* gene was reported for the first time in Egypt by Shebl et al. [18], who detected the *mecC* gene in three PCR-tested MRSA isolates, representing 6% of the total isolates. This study was conducted in the largest university hospital in Egypt, which is the target of many patients from different rural and urban areas.

Remarkably, 21/118 (17.8%) isolates of *S. aureus* were phenotypically resistant but did not carry either the *mecA* or *mecC* genes. Of these 21 isolates, six had a MIC of 4 μg/mL with oxacillin BMD, while the MIC of the remaining 15 isolates ranged from 256 μg/mL to ≥512 μg/mL. It is important to clarify that the absence of the *mecA* and *mecC* genes is no longer used as a reliable marker to exclude MRSA [43]. In the current study, the existence of phenotypically MRS isolates with negative *mecA* and *mecC* genes can be attributed to mutations in genes encoding PBP [51] or by the presence of hyper-β-lactamase-producing strains, which were termed borderline oxacillin-resistant *S. aureus* (BORSA); these strains show low borderline resistance to oxacillin [52]. BORSA is not a carrier of modified PBP2a encoded by either the *mecA* or *mecC* genes [53]. In this study, the six isolates with a low MIC of 4 μg/mL may have been BORSA isolates. Further studies are required to specifically characterize the mechanism of oxacillin resistance in our *mecA*- and *mecC*-negative isolates.

Besides *S. aureus*, four different species of CoNS were detected in our research, where *S. epidermidis* was the most prevalent. *S. epidermidis* has been shown to be the most frequently isolated CoNS in numerous surveys [54,55,56]. In disparity, some surveys have identified *S. capitis* [56]. In this study, the second-most frequent CoNS was S. *haemolyticus*, which was observed mainly in CSF samples; this was in accordance with Singh et al. [57]. We detected that *S. saprophyticus* was the least prevalent species, which was detected mainly in urine samples. According to published research, *S. saprophyticus* is a frequently isolated CoNS and a common cause of urinary tract infection [57,58,59]. The distribution of different species may be influenced by patient features that affect colonization, as well as how well each species adapts to environmental factors such as biocides and antimicrobials [57].

Over the past few decades, oxacillin resistance in CoNS isolates has significantly increased. More than 80% of our CoNS isolates showed resistance to oxacillin, with the highest MR in *S. haemolyticus,* which is supported by results from other centers with resistance rates up to 90% [60]. Consequently, the need for more expensive and perhaps more toxic therapeutic medicines [61].In the current study, all CoNS isolates were susceptible to vancomycin and linezolid; these findings are in accordance with Singh et al. [57]. Patients with MRCoNS infections may receive these medications as part of their treatment; however, their use as an empirical therapy must be avoided since excessive use of these antibiotics might lead to the development of glycopeptide and oxazolidinone resistance [60,62].

Interestingly, in the present study, 13 out of 43 CoNS isolates (30.2%) carried the *mecA* gene but were susceptible to cefoxitin, as demonstrated by DD testing; they showed cefoxitin resistance in VITEK2 testing and oxacillin resistance in BMD and VITEK2 testing. Based on the CLSI guidelines, the cefoxitin DD test is currently recommended as a surrogate for the oxacillin DD test [22]. This may be due to the fact that cefoxitin is a potent promotor of the *mecA* gene that is less affected than oxacillin by the hyperproduction of penicillinase [63,64]. However, over the last decade, unique *S. aureus* strains have been identified and categorized as oxacillin-susceptible MRSA (OS-MRSA); these strains possess a *mecA* gene but are phenotypically sensitive to cefoxitin and oxacillin [65,66]. Little is known about CoNS that demonstrate this phenomenon (OS-CoNS), though they have been reported by a recent study in the UK [67]. As far as we know, the present study is the first report of OS-CoNS strains in Egypt.

These unique strains are highly heterogeneous and have been shown to be “inducible oxacillin resistant” [66]. Several studies reported the changing of these highly heterogeneous OS-MRSAs into homogeneously oxacillin-resistant strains after exposing them to different concentrations of oxacillin and cefotaxime [65,68]. This may explain why the 13 CoNS isolates in our study were susceptible to cefoxitin DD, though later, they became cefoxitin- and oxacillin-resistant in the VITEK2 system and in oxacillin BMD. In agreement with this, other studies reported OS-MRSA isolates’ resistance to oxacillin and cefoxitin in the VITEK2 system [69,70]. This unique phenotypic–genotypic disparity has been suggested to be related to mutations in the sites of nucleotide repeats within the *mecA* gene, making such strains phenotypically susceptible to oxacillin. These strains become resistant after antibiotic exposure by simple and relatively frequent point mutation to restore gene function [71]. Therefore, using β-lactam drugs to treat such strains may lead to the failure of therapy as oxacillin resistance is induced in vivo [72].

One of the most striking findings in the current study was the high level of methicillin resistance among our isolates of *S. aureus* and CoNS, since 82.2% of our *S. aureus* isolates and 95.3% of CoNS isolates were methicillin-resistant and carrying the *mecA* gene. In previous studies carried out in other Egyptian cities, the prevalence of methicillin resistance ranged from 44% to 88.2% in HA-*S. aureus* [73,74], and from 38.8% to 75% in HA-CoNS [74,75]. Our data are consistent with a previous report comparing the rates of antibiotic resistance between the countries of the Arab League, in which Egypt showed the highest prevalence of methicillin resistance in *S. aureus* among 19 Arabic countries [76]. All these data highlight a serious problem in Egyptian hospitals. The high level of resistance encountered in our hospital can be attributed to the unrestricted use of antibiotics and lack of resources for infection control, resulting in the lax implementation of infection prevention measures, which contributes to increasing the rate of resistance to HA-*Staphylococcal* infection.

Among the phenotypic techniques used in this study to identify methicillin resistance in *S. aureus* isolates the cefoxitin disc diffusion test exhibited the best diagnostic performance, with 100% sensitivity compared to *mecA* PCR. Similar results were reported by many researchers who used PCR as a reference method [77,78,79], while Perazzi et al. and Martins et al. [80,81] reported lower sensitivities of 80% and 91.3%, respectively. We detected that cefoxitin disc was superior to oxacillin, consistent with previous reports [82,83].

Regarding CoNS isolates, VITEK2 Cefoxitin had the best diagnostic performance among all phenotypic methods used for the detection of methicillin resistance, with a sensitivity of 97.56%, followed by VITEK2 oxacillin (90.24%); BMD oxacillin (87.80%), while cefoxitin DD showed the lowest sensitivity (68.29%). According to our study, Graham et al. [84] measured oxacillin sensitivity using oxacillin DD and oxacillin MIC by E-test and found that these methods are insufficient to identify methicillin resistance. Contrary to our findings, Shrestha et al. [85] showed high sensitivity (95.4%) to cefoxitin DD; moreover, similar results have also been reported by Secchi et al. and Bhatt et al. [86,87].

## 5. Conclusions

No *mecC*-harboring *Staphylococci* were isolated in this study. However, they were detected in a minimal non-alarming percentage in two previous Egyptian studies. The lack of data on the prevalence of *mecC*-carrying MRSA isolates from Egypt may be due to the low prevalence of this resistance mechanism or the limited number of performed studies. However, it is important to clarify that the absence of the *mecA* and *mecC* genes is no longer used as a reliable marker to exclude methicillin resistance. Therefore, further studies are required to specifically characterize the mechanism of oxacillin resistance in our *mecA* and *mecC* negative isolates. On the other hand, the high rate of methicillin resistance in our *Staphylococci* is worrisome, raising the alarm towards revising the antibiotic policy and infection prevention and control protocols in our hospitals. In addition, this is the first Egyptian study that has shed light on *mecA*-positive OS-CoNS strains and the need for increased molecular epidemiological studies for a better understanding of the impact of these strains in human infections, especially HA infections, and the best laboratory methods for their accurate diagnosis.

## Figures and Tables

**Table 1:** Comparison between *S. aureus* and CoNS according to basic characteristics of the patients.

	*S. aureus* (n = 118)	CoNS (n = 43)	Test of Sig.	*p*
Age				
Range (years)	1.5–75	1–76	*t* = 0.150	0.881
Mean (±SD)	33.46 (±19.4)	32.9 (±24.7)		
Gender				
Male	66 (55.9%)	22 (51.2%)	χ^2^ = 0.289	0.591
Female	52 (44.1%)	21 (48.8%)		
Sample type (total number)				
Blood (153)	46 (39%)	20 (46.5%)	χ^2^ = 0.738	0.390
CSF (50)	0 (0%)	17 (39.5%)	χ^2^ = 52.159 *	^FE^ *p* < 0.001 *
Urine (92)	15 (12.7%)	6 (14%)	χ^2^ = 0.043	0.836
Wound (111)	45 (38.1%)	0 (0%)	χ^2^ = 22.760 *	<0.001 *
Sputum (94)	12 (10.2%)	0 (0%)	χ^2^ = 4.725 *	^FE^ *p* = 0.037 *

SD: Standard deviation; t: Student *t*-test; χ^2^: Chi-square test; ^FE^: Fisher Exact; *p*: *p*-value; CoNS: coagulase-negative *Staphylococci*; CSF, cerebrospinal fluid; *: Statistically significant at *p* ≤ 0.05.

**Table 2:** Results of *S. aureus* and CoNS surveillance, including (Month/Year of isolation, number of collected samples, and number of *S. aureus* and CoNS isolates) during the study period.

Season (No. of *Staphylococci*) (% of Total Samples)		Month/Year	No. of Collected Samples	No. of *S. aureus*	No. of CoNS
Spring (n = 47) (33.1%)		March 2021	55	14	-----
		April 2021	55	15	7
		May 2021	32	10	1
Summer (n = 43) (35.8%)		June 2021	42	10	6
		July 2021	31	5	4
		August 2021	46	14	4
Autumn (n = 42) (32.1%)		September 2021	58	15	5
		October 2021	36	5	4
		November 2021	37	8	5
Winter (n = 13) (15.3%)		December 2021	36	4	----
		January 2022	25	2	3
		February 2022	24	4	----
		March 2022	23	12	4
Total			500	118	43
χ^2^	*p*				
11.514 *	0.009 *				

CoNS: coagulase-negative Staphylococci; B: blood; CSF: cerebrospinal fluid; U: urine; W: wound; S: sputum; χ^2^: Chi-square test; *p*: *p*-value for comparing between the different studied groups; *: Statistically significant at *p* ≤ 0.05.

**Table 3:** Distribution of CoNS species isolated from different clinical samples.

	CoNS Species					χ^2^	^MC^ *p*
	*S. epidermidis*	*S. haemolyticus*	*S. hominis*	*S. saprophyticus*	Total		
Sample type							
Blood	10 (58.8%)	5 (31.3%)	5 (100%)	0 (0%)	20 (100%)	26.750 *	<0.001 *
CSF	7 (41.2%)	10 (62.5%)	0 (0%)	0 (0%)	17 (100%)		
Urine	0 (0.0%)	1 (6.3%)	0 (0%)	5 (100%)	6 (100%)		
	17 (39%)	16 (37%)	5 (12%)	5 (12%)	43 (100%)		

CoNS: coagulase-negative Staphylococci; *S. epidermidis*: Staphylococci epidermidis; *S. haemolyticus*: Staphylococci haemolyticus; *S. hominis*: Staphylococci hominis; *S. saprophyticus*: Staphylococci saprophytics: χ^2^: Chi-square test; ^MC^: Monte Carlo; *p*: *p*-value for comparing between the different studied groups; *: Statistically significant at *p* ≤ 0.05.

**Table 4:** Antimicrobial resistance patterns among *S. aureus* isolates.

Pattern Code	Antimicrobial Resistance Pattern	MAR Index	Number of MRSA Isolates (n = 118)
S II	P, FOX	0.16	2 (1.7%)
S III a	P, FOX, E	0.25	3 (2.5%)
S III b	P, FOX, C	0.25	5 (4.2%)
S III c	P, FOX, DA	0.25	4 (3.4%)
S III d	P, FOX, RD	0.25	5 (4.2%)
S IV a	P, FOX, E, C	0.33	9 (7.6%)
S IV b	P, FOX, E, DA	0.33	4 (3.4%)
S IV d	P, FOX, E, CIP	0.33	5 (4.2%)
S IV e	P, FOX, C, CIP	0.33	3 (2.5%)
S V a	P, FOX, E, C, DA	0.42	5 (4.2%)
S V b	P, FOX, E, C, CIP	0.42	4 (3.4%)
S V c	P, FOX, C, CIP, DA	0.42	5 (4.2%)
S V d	P, FOX, C, CIP, CN	0.42	3 (2.5%)
S V e	P, FOX, TE, C, DA	0.42	2 (1.7%)
S V f	P, FOX, CIP, SXT, DA	0.42	4 (3.4%)
S VI a	P, FOX, E, C, DA, CIP	0.5	2 (1.7%)
S VI b	P, FOX, E, C, DA, CN	0.5	3 (2.5%)
S VI c	P, FOX, E, C, DA, SXT	0.5	4 (3.4%)
S VI d	P, FOX, C, E, CIP, SXT	0.5	2 (1.7%)
S VI e	P, FOX, CN, C, RD, SXT	0.5	2 (1.7%)
S VI f	P, FOX, SXT, C, TE, E	0.5	1 (0.8%)
S VI g	P, FOX, SXT, CIP, TE, RD	0.5	2 (1.7%)
S VII a	P, FOX, E, C, CIP, SXT, CN	0.58	3 (2.5%)
S VII b	P, FOX, E, C, DA, CIP, CN	0.58	4 (3.4%)
S VII c	P, FOX, E, C, DA, CIP, TE	0.58	2 (1.7%)
S VII d	P, FOX, E, C, DA, CIP, SXT	0.58	4 (3.4%)
S VII e	P, FOX, E, C, DA, CIP, RD	0.58	3 (2.5%)
S VII f	P, FOX, E, C, DA, CIP, CN	0.58	2 (1.7%)
S VIII a	P, FOX, E, C, DA, CIP, CN, SXT	0.67	4 (3.4%)
S VIII b	P, FOX, E, C, DA, CIP, CN, TE	0.67	4 (3.4%)
S VIII c	P, FOX, E, C, DA, CIP, CN, RD	0.67	3 (2.5%)
S IX a	P, FOX, E, C, DA, CIP, CN, SXT, RD	0.75	1 (0.8%)
S IX b	P, FOX, E, C, DA, CIP, CN, SXT, TE	0.75	4 (3.4%)
S IX c	P, FOX, E, C, DA, CIP, CN, RD, TE	0.75	1 (0.8%)
S X a	P, FOX, E, C, DA, CIP, CN, TE, RD, SXT	0.83	4 (3.4%)

S: *Staphylococci*; II-XII: groups according to the number of resistant antibiotics; MAR: multiple antibiotic resistance; MRSA: Methicillin-resistant *Staphylococcus aureus*; P: Penicillin, FO: Cefoxitin; VA: Vancomycin; CN: Gentamicin; E: Erythromycin; TE: Tetracycline, CIP; Ciprofloxacin, DA; Clindamycin, SXT; Trimethoprim-sulfamethoxazole, C; Chloramphenicol, RD; Rifampin, LZD; Linezolid: a–f; different combinations of antibiotics for each Latin number group.

**Table 5:** Prevalence of methicillin resistance among *Staphylococcal* isolates detected by genotypic method (PCR).

Staphylococcus Species	Total no. of Isolates	No. (%) of mecA PCR-Positive Isolates	No. (%) of mecC PCR-Positive Isolates
*S. aureus*	118	97 (82.2)	-
*S. epidermidis*	17	16 (94)	-
*S. hemolyticus*	16	15 (93.7)	-
*S. hominis*	5	5 (100)	-
*S. saprophyticus*	5	5 (100)	-

**Table 6:** Sensitivity and specificity of different phenotypic methods compared to the genotypic method (PCR) in detecting methicillin resistance in *Staphylococci*.

***S. aureus*** **(n = 118)**							
	**PCR *mecA***		**Sensitivity**	**Specificity**	**PPV**	**NPV**	**Accuracy**
	**Negative (n = 21)**	**Positive (n = 97)**					
Cefoxitin DD resistance VITEK2 cefoxitin resistance BMD oxacillin resistance							
Negative	0	0	100.0	0.0	82.20	–	82.20
Positive	21	97					
VITEK2 oxacillin resistance							
Negative	21	14	85.57	100.0	100.0	60.0	88.14
Positive	0	83					
**CoNS species (n = 43)**							
	**PCR *mecA***		**Sensitivity**	**Specificity**	**PPV**	**NPV**	**Accuracy**
	**Negative (n = 2)**	**Positive (n = 41)**					
Cefoxitin DD resistance							
Negative	2	13	68.29	100.0	100.0	13.33	69.77
Positive	0	28					
VITEK2 oxacillin resistance							
Negative	2	4	90.24	100.0	100.0	33.33	90.70
Positive	0	37					
VITEK2 cefoxitin resistance							
Negative	2	1	97.56	100.0	100.0	66.67	97.67
Positive	0	40					
BMD oxacillin resistance							
Negative	2	5	87.80	100.0	100.0	28.57	88.37
Positive	0	36					

CoNS: coagulase-negative *Staphylococci*; DD: disc diffusion; BMD: broth microdilution; PPV: Positive predictive value; NPV: Negative predictive value.

## Data Availability

Data are available from the corresponding author upon request.

## Supplementary Materials

The following supporting information can be downloaded at: https://www.mdpi.com/article/10.3390/pathogens12040556/s1, Table S1: The sequence of the used primers, product size and PCR protocol of *mecA* and *mecC* genes.; Table S2: Different bacterial and fungal isolates distribution in the collected clinical samples.
