# Supplementary material for: Phenotypic and Genotypic Characterization of Methicillin Resistance in Staphylococci Isolated from an Egyptian University Hospital
Source: Pathogens. 2023 Apr 5;12(4):556. doi: 10.3390/pathogens12040556 (PMC10143866; doi:10.3390/pathogens12040556)
Supplement: Supplementary file 1 [file pathogens-12-00556-s001.zip › pathogens-2264410-supplementary.pdf]

Supplementary

Table S1. The sequence of the used primers, product size and PCR protocol of *mecA* and *mecC* genes.

SCCmec type	Nucleotide sequence (5′-3′)	PCR product Size (bp)	Ref	PCR protocol
mecA	F: GTAGAAATGACTGAACGTCCGATAA<br>R: CCAATTCCACATTGTTTCGGTCTAA	310	[87]	Initial denaturation: 95°C for 1 min<br>Amplification (30 cycles of):<br>- Denaturation: 95°C for 30 s<br>- Annealing: 50°C for 30 s<br>- Extension: 72°C for 60 s<br>Final extension: 72°C for 4 min<br>The amplified product was identified by electrophoresis on 1.5% agarose gels using 100 bp DNA ladder.
mecC	F: GCTCCTAATGCTAATGCA<br>R: TAAGCAATAATGACTACC	304	[15]	Initial denaturation: 95°C for 2 min<br>Amplification (30 cycles of):<br>- Denaturation: 94°C for 30 s<br>- Annealing: 50°C for 30 s<br>- Extension: 72°C for 30 s<br>Final extension: 72°C for 4 min<br>The amplified product was identified by electrophoresis on 1.5% agarose gels using 100 bp DNA ladder.

F, forward; R, reverse; bp, base pair; Ref, reference

Table S2. Different bacterial and fungal isolates distribution in the collected clinical samples

Isolated organism	Number	Sample
**Gram positive isolates**	210	
Staphylococcus aureus	118	Blood- urine-sputum-wound
CoNS	43	Blood- urine-CSF
Enterococcus spp	30	Blood- urine-wound
Streptococcus spp	19	Sputum-CSF
**Gram negative isolates**	307	
E.coli	82	Blood- urine-wound
Klebsiella spp	73	Blood- urine-sputum-wound-CSF
Acinetobacter spp	49	Blood- urine-sputum-wound-CSF
Pseudomonas spp	87	Blood- urine-sputum-wound
Enterobacter spp	4	Sputum-wound
Stenotrophomonas spp	12	Urine-sputum-wound
**Fungi**		
Candida spp	36	Urine-sputum-wound
Total isolates	553	

CoNS: coagulase-negative Staphylococci; CSF, cerebrospinal fluid; spp: species; *E.coli : Escherichia coli*
